# Inhibitory effects of aspirin-triggered resolvin D1 on spinal nociceptive processing in rat pain models

**DOI:** 10.1186/s12974-016-0676-6

**Published:** 2016-09-02

**Authors:** Pongsatorn Meesawatsom, James Burston, Gareth Hathway, Andrew Bennett, Victoria Chapman

**Affiliations:** 1 Arthritis Research UK Pain Centre, Queen’s Medical Centre, School of Life Sciences, University of Nottingham, Nottingham, NG7 2UH UK; 2 FRAME Alternatives Laboratory, Queen’s Medical Centre, School of Life Sciences, University of Nottingham, Nottingham, NG7 2UH UK

**Keywords:** AT-RvD1, Pain, Inflammation, Osteoarthritis, Electrophysiology

## Abstract

**Background:**

Harnessing the actions of the resolvin pathways has the potential for the treatment of a wide range of conditions associated with overt inflammatory signalling. Aspirin-triggered resolvin D1 (AT-RvD1) has robust analgesic effects in behavioural models of pain; however, the potential underlying spinal neurophysiological mechanisms contributing to these inhibitory effects in vivo are yet to be determined. This study investigated the acute effects of spinal AT-RvD1 on evoked responses of spinal neurones in vivo in a model of acute inflammatory pain and chronic osteoarthritic (OA) pain and the relevance of alterations in spinal gene expression to these neurophysiological effects.

**Methods:**

Pain behaviour was assessed in rats with established carrageenan-induced inflammatory or monosodium iodoacetate (MIA)-induced OA pain, and changes in spinal gene expression of resolvin receptors and relevant enzymatic pathways were examined. At timepoints of established pain behaviour, responses of deep dorsal horn wide dynamic range (WDR) neurones to transcutaneous electrical stimulation of the hind paw were recorded pre- and post direct spinal administration of AT-RvD1 (15 and 150 ng/50 μl).

**Results:**

AT-RvD1 (15 ng/50 μl) significantly inhibited WDR neurone responses to electrical stimuli at C- (29 % inhibition) and Aδ-fibre (27 % inhibition) intensities. Both wind-up (53 %) and post-discharge (46 %) responses of WDR neurones in carrageenan-treated animals were significantly inhibited by AT-RvD1, compared to pre-drug response (*p* < 0.05). These effects were abolished by spinal pre-administration of a formyl peptide receptor 2 (FPR2/ALX) antagonist, butoxy carbonyl-Phe-Leu-Phe-Leu-Phe (BOC-2) (50 μg/50 μl). AT-RvD1 did not alter evoked WDR neurone responses in non-inflamed or MIA-treated rats. Electrophysiological effects in carrageenan-inflamed rats were accompanied by a significant increase in messenger RNA (mRNA) for chemerin (ChemR23) receptor and 5-lipoxygenase-activating protein (FLAP) and a decrease in 15-lipoxygenase (15-LOX) mRNA in the ipsilateral spinal cord of the carrageenan group, compared to controls.

**Conclusions:**

Our data suggest that peripheral inflammation-mediated changes in spinal FLAP expression may contribute to the novel inhibitory effects of spinal AT-RvD1 on WDR neuronal excitability, which are mediated by FPR2/ALX receptors. Inflammatory-driven changes in this pathway may offer novel targets for inflammatory pain treatment.

**Electronic supplementary material:**

The online version of this article (doi:10.1186/s12974-016-0676-6) contains supplementary material, which is available to authorized users.

## Background

Untreated pain remains a major clinical problem, and there is a need for the identification of novel therapeutic approaches for both acute and chronic pain states. Sustained activation of the pain pathways is underpinned by the actions of peripheral and spinal inflammatory cells, peripheral sensitization of nerve terminals and the plasticity of the spinal neuronal circuits activated [1, 2]. It is established that nociceptive afferent barrage leads to changes within the spinal cord, including the relatively quick up- and down-regulation of genes for various enzymatic pathways [3–5], which may act to promote nociceptive responses and drive hyperalgesia or act to counteract these events.

Specialised proresolving mediators (SPMs) such as the essential fatty acid-derived lipoxins, resolvins, protectins and maresins [6, 7] have robust inhibitory effects on inflammatory signalling pathways. This has been particularly well evidenced for the D-series resolvins, specifically resolvin D1 (RvD1 or 17S-RvD1) and its isomer, aspirin-triggered RvD1 (AT-RvD1 or 17R-RvD1), which are SPMs derived from the polyunsaturated fatty acid docosahexaenoic acid (DHA) [8]. SPMs are generated after an overt inflammatory insult to promote resolution [6], and this is achieved by the inhibition of pro-inflammatory cytokine production and shortening of the interval between inflammation and resolution by inhibiting neutrophil infiltration and stimulating macrophage phagocytosis [6]. Endogenous synthesis of the D-series resolvins is via the 15-lipoxygenase (15-LOX)-mediated conversion of DHA to hydroperoxy intermediates (see Additional file 1: Figure S1). Synthesis of AT-RvD1 is enhanced by aspirin [8] via the acetylation of a serine residue on cyclooxygenase-2 (COX-2), to permit enzymatic conversion of DHA to a 17R-hydroperoxy intermediate, prior to 5-lipoxygenase (5-LOX)-mediated RvD1/AT-RvD1 formation. RvD1 and AT-RvD1 are rapidly metabolised, predominantly by 15-prostaglandin dehydrogenase to 8-oxo and 17-oxo metabolites [9]. A metabolically more stable analogue of resolvin D1 has been developed with similar potency in reducing neutrophil infiltration and increasing macrophage phagocytosis [10]. The biological effects of RvD1 and AT-RvD1 have been attributed to the G-protein-coupled receptor GPR32 and formyl peptide receptor 2 (FPR2/ALX), utilising Gi and possibly Gq as signal transductions [11, 12]. Both receptors are expressed in human tissue, but GPR32 is not yet identified in rodents [13].

Despite their rapid degradation, locally administered RvD1 and AT-RvD1 have analgesic effects in various animal models of pain [13–17]. In carrageenan-induced inflammatory pain, intraplantar administration of RvD1 attenuated paw oedema and heat hyperalgesia [14]. Single intrathecal administration of RvD1 or AT-RvD1 rapidly decreased heat pain thresholds and reduced mechanical hypersensitivity in behavioural tests in the carrageenan model [13, 14]. The spinal neuronal mechanisms underlying these neurophysiological effects on pain behaviour have yet to be elucidated.

The future exploitation of this potential novel class of analgesics requires a comprehensive understanding of their sites and mechanisms of action and the conditions under which inhibitory effects are evident. Here, we characterise the neurophysiological mechanism effects underpinning the effects of AT-RvD1 on spinal nociceptive processing using in vivo electrophysiology in two models of pain. Acute effects of spinal administration of AT-RvD1 on evoked spinal neuronal responses were characterised in the carrageenan-induced model of inflammatory pain and monosodium iodoacetate (MIA) model of chronic joint pain. To advance mechanistic understanding of this system in the spinal cord, gene expression of the known resolvin receptors in the rodent (FPR2/ALX and chemerin (ChemR23) receptor) and relevant enzymatic pathways, in particular 15-LOX and 5-LOX, were quantified in the model of inflammatory pain, compared to the relevant control group.

## Methods

### Animals

Male Sprague Dawley rats (*n* = 84) were purchased from Charles River, UK. The initial weight ranges were 200–250 g and 175–220 g on the day of carrageenan and MIA induction, respectively. Studies were carried out in accordance with the UK Home Office Animals (Scientific Procedures) Act (1986) and followed the guidelines of the International Association for the Study of Pain [18] and were approved by the local ethical review board at the University of Nottingham. Rats were group housed at the Bio Support Unit, University of Nottingham, in open cages and fed ad libitum. In accordance with the ARRIVE guidelines [19], full details of the group sizes for the different studies and experimental endpoints are in Additional file 1: Table S1.

### Induction of pain models

Under brief anaesthesia (isoflurane 2.5–3 % in O_2_ 1 l/min), rats were injected with either intraplantar 2 % λ-carrageenan 100 μl (Sigma, UK) or vehicle (0.9 % saline) into the glabrous surface of the left hind paw [20] or intra-articular 1 mg/50 μl of MIA (Sigma, UK) or vehicle injection via infra-patella ligament [21].

### Pain behaviour measurements

Pain behaviour was quantified as previously described [21, 22] in a blinded fashion at baseline, 2 and 8 h post carrageenan injection and twice a week post MIA injection for 28 days. Weight bearing asymmetry, reflecting the change in weight bearing from the ipsilateral hind limb to the contralateral hind limb, was measured using an incapacitance tester (Linton Instrumentation, UK). Paw withdrawal thresholds (PWTs) of both hind paws were assessed using the up-down method [23] with von Frey monofilaments with a range of bending forces (1, 1.4, 2, 4, 6, 8, 10, 15 and 26 g), starting for 4 g. Once a withdrawal reflex was observed, the next descending monofilament was applied to retest until no response was elicited. PWT was determined as the lowest force of monofilament which evoked a paw withdrawal reflex [24].

### In vivo spinal electrophysiology

Single-unit in vivo electrophysiology recording of deep dorsal horn wide dynamic range (WDR) neurones was performed, as previously described [25] on the day following carrageenan injection or on days 28–32 post MIA injection.

Rats were anaesthetised with isoflurane (3 % induction, 2 % surgery, 1.5–1.75 % maintenance) in 0.6 l/min N_2_O and 0.3 l/min O_2_, and a tracheal cannula was inserted. Rats were placed in a stereotaxic frame, and a laminectomy was performed to expose the L4–L5 region of the spinal cord receiving the input from the hind paw. The size of the laminectomy was kept to a minimum; only the narrow centre strip of tissue and vertebrae was removed, leaving the edges of the vertebrae covered by the surrounding paraspinal muscles to form a natural well-retaining applied fluid. The spinal column was held rigid by clamps caudal and rostral to the exposed section. Core body temperature was maintained (36.5–37 °C) via a homeothermic heated blanket linked to a rectal probe (Harvard Instruments, UK). A glass-coated tungsten microelectrode was slowly lowered into the ipsilateral side of the dorsal horn using a SCAT-01 Microdrive (Digitimer, UK) in 10-μm steps. Electrical activity was amplified, filtered by a Neurolog system (Digitimer, UK), digitised by CED Micro1401 (Cambridge Electronic Design, Cambridge, UK) and captured/analysed by Spike 2 version 6.05 software (Cambridge Electronic Design, UK). Single neurones located between 500 and 1000 μm from the surface of the spinal cord were recorded. Neurones that responded to both innocuous mechanical stimuli (gently tapping) and noxious stimuli (pinching) were defined as being WDR. Responses of WDR neurones following a train of 16 (0.5 Hz, 2-ms pulse width) consecutive transcutaneous electrical stimuli delivered at the centre of the area on the hind paw eliciting the WDR neurone responses were characterised. The advantages of transcutaneous electrical stimulation include controlled and reproducible stimulus delivery in the presence of tissue inflammation [26], quantification of distinct peripheral fibre group-evoked responses and the bypassing of peripheral transduction process to ensure that any changes in neuronal responses are centrally mediated [27]. All selected neurones had a clear short-latency Aβ-fibre-evoked response followed by a C-fibre-evoked response. A single electrical stimulus was applied at increasing amplitude in 0.1-mA steps; when a single action potential was elicited in a 90–300-ms post-stimulus range, the stimulus amplitude was taken as the C-fibre threshold and the latency were recorded. Aβ-fibre threshold and latency were measured in the same manner but with a 0.01-mA amplitude increment and in a 0–20-ms post-stimulus range. Responses were elicited at three times (3×) the threshold for C-fibres and then 3× the threshold for Aβ fibres, and post-stimulus histograms were built. Note that C-fibre stimulus intensity also activates Aβ and Aδ fibres which will modulate WDR neurone responses. The Aβ-fibre-evoked responses were taken as the action potential number recorded 0–20 ms after the electrical stimulus, Aδ-fibre-evoked responses were taken as the action potential number recorded 20–90 ms after the electrical stimulus, and C-fibre-evoked responses were taken as the action potential number recorded 90–300 ms after the electrical stimulus. The remaining neuronal response (300–800 ms post stimulus) was defined as the post-discharge (PD) of the neurone. The input (non-potentiated response) was calculated as the number of action potentials in C-fibre and PD bands produced by the first stimulation (initial baseline response) multiplied by the total number of stimuli (16). Wind-up (WU, potentiated response) [28] was calculated as the difference between the total number of action potentials in C-fibre and PD bands produced by the train of 16 stimuli minus the input. Cycles of the electrical stimulations were carried out in 15-min intervals.

### Pharmacological studies

Following stable control-evoked neuronal responses (<10 % variation of C-fibre responses), drugs were applied topically to the surface of the exposed L4-5 segments of the spinal cord via a Hamilton syringe in 50-μl volume. Concentrations of AT-RvD1 and butoxy carbonyl-Phe-Leu-Phe-Leu-Phe (BOC-2), a FPR2/ALX receptor antagonist [29], were based on previous studies [13, 30]. AT-RvD1 and RvD1 have similar chemical structures; however, AT-RvD1 is more resistant to enzymatic inactivation by 15-hydroxyprostaglandin dehydrogenase (15-PGDH) [9]. In the carrageenan study, saline- or carrageenan-pre-treated rats received one of the following spinal treatments: AT-RvD1 (Cayman Chemical, USA) 15 ng in 50 μl PBS (phosphate-buffered saline); vehicle (50 μl PBS); BOC-2 (Phoenix Pharmaceuticals, Inc., USA) 50 μg in 50 μl 3 % Tween 80 + 0.5 % ethanol in saline; BOC-2 50 μg in 50 μl 3 % Tween 80 + 0.5 % ethanol in saline for 15 min and then AT-RvD1 at 15 ng. In the MIA study, saline- and MIA-treated rats received spinal AT-RvD1 cumulatively (15 ng and 150 ng /50 μl) and then spinal morphine sulphate (Queen’s Medical Centre Pharmacy, Nottingham, UK) 1 μg/50 μl in PBS vehicle. Previous drug solution was removed from the spinal cord between treatments. Effects of treatments on electrically evoked responses of neurones were followed for 60 min post treatment. Full details of the group sizes are provided in Additional file 1: Table S1.

### Gene expression study

In separate cohorts of intraplantar saline- (*n* = 6) and carrageenan-injected rats (*n* = 5), pain behaviour was assessed as previously described. Rats were sacrificed by cranial concussion and decapitated (Schedule 1 of the Animal (Scientific Procedure) Act 1996) at 30 h post carrageenan induction. The lumbar enlargement (L4–L6) of the spinal cord was dissected free and the ipsilateral dorsal horn quadrant was frozen on dry ice and stored at −80 °C until use. Note that the collected samples are a mixture of all cell types, e.g. neurones, microglia and astrocytes. After treating with 2 ml ice-cold Tri reagent (Sigma Aldridge, UK), total RNA was extracted and purified from the tissue samples according to the manufacturer’s instructions. Complementary DNA (cDNA) was synthesised by reverse transcription from 500 ng total RNA using SuperScript III (Invitrogen, UK) reverse transcriptase according to the manufacturer’s instructions. Reactions were incubated at 25 °C for 10 min, 37 °C for 50 min and followed by 70 °C for 15 min to terminate the reaction. Gene expression quantification was performed as previously described [31, 32]. Genes examined relative to β-actin were FPR2/ALX, ChemR23, 5-LOX, 15-LOX, COX-2, 5-lipoxygenase-activating protein (FLAP) and interleukin-10 (IL-10). Primers and probes were based on a previous study [5] or designed by Primer Express 3.0 software and synthesised by MWG Biotech (Germany); sequences are in Additional file 1: Table S2.

### Macroscopic assessments

The circumference of ipsilateral paw was measured at baseline and 24 h post carrageenan injection as previously described [20, 33]. Ipsilateral knee joints of rats in the MIA study were collected at the end of the study and were disarticulated. Macroscopic scoring of knee joint pathology was based on a previously reported scoring system [34]. The severity of the pathology of the cartilage surface was graded as follows: 0 = normal appearance, 1 = slight yellowish discoloration of the chondral surface, 2 = little cartilage erosions in load-bearing areas, 3 = large erosions extending down to the subchondral bone, and 4 = large erosions with large areas of subchondral bone exposure. Six compartments were scored including medial femoral condyle, lateral femoral condyle, medial tibial plateau, lateral tibial plateau, femoral groove and patella and then were combined to give a total score (possible maximum score 24). Abnormal growth of bone was recorded as osteophyte presentation. The experimenter was blinded for the induction procedure.

### Statistical analysis

GraphPad Prism 6.05 (GPP 6.05; GraphPad Software, Inc, San Diego, CA) was used for statistical analyses and graphical presentation. Data were excluded from the statistical analysis where outliers were identified by Grubb’s test at *α* = 0.05. Results are expressed as mean ± standard error of mean (SEM). Kolmogorov-Smirnov test was used to determine whether data are normally distributed. Percentage of weight (wt) bearing asymmetry was calculated from the formula [(Contralateral wt − Ipsilateral wt) / (Contralateral wt + Ipsilateral wt)] × 100. Pain behaviour data were analysed with a two-way analysis of variance (ANOVA) with Sidak post hoc test. Differences in paw circumference between carrageenan and saline and chondropathy scores between MIA versus saline were analysed using a Mann-Whitney *U* test. Raw data from the electrophysiological study using the carrageenan model were analysed with paired *t* tests or Wilcoxon tests. In the MIA study, raw data from the electrophysiological study were compared to baseline using repeated measure ANOVA followed by Sidak post hoc test or Friedman statistics followed by Dunn’s post hoc test. Percentages of maximal inhibition were calculated versus baseline (pre-drug responses) and were compared between groups using Kruskal-Wallis test with Dunn’s post hoc test. Statistical analysis of gene expression data was performed using a Mann-Whitney *U* test. Statistical significance is considered where *p* value is ≤0.05 for all comparisons.

## Results

### Carrageenan-mediated hind paw inflammation resulted in alterations in WDR neurone responses

**Fig. 1:**
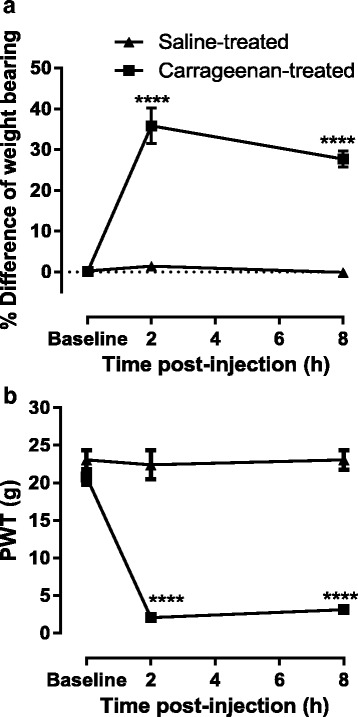
Carrageenan-evoked pain behaviour and paw inflammation. Intraplantar injection of 2 % λ-carrageenan significantly increased weight bearing asymmetry (**a**) and decreased mechanical PWT (**b**) from 2 to 8 h post injection. ****p < 0.0001 versus the saline-treated group, two-way repeated measure ANOVA with Sidak post hoc test. Saline *n* = 15 and carrageenan *n* = 38

Initial paw circumferences of saline- and carrageenan-treated rats were comparable, 25 ± 0.3 and 26 ± 0.2 mm, respectively. Carrageenan-treated rats exhibited robust weight bearing asymmetry and lowered PWTs at 2 and 8 h post injection, compared to saline-treated rats (Fig. 1a, b). Intraplantar injection of carrageenan also produced a profound paw swelling, evident at 1–2 h and significant at 24 h post injection compared to saline-treated rats (carrageenan 33 ± 0.28 mm; saline 25.8 ± 0.29 mm, *p* < 0.0001).

WDR neurones (*n* = 53) were characterised in a total of 53 rats (saline-treated *n* = 15 and carrageenan-treated *n* = 38) at 24 h after intraplantar injection of saline or carrageenan (Table 1). The mean depths of neurones of all groups were within the 500–1000-μm range, corresponding to laminae V–VI (Table 1). Comparison of thresholds of evoked responses of WDR neurones revealed higher thresholds for Aβ- and C-fibre-evoked responses in the carrageenan-treated group (Table 1). Aβ- and C-fibre latencies and magnitudes of evoked firing of WDR neurones were not altered in the carrageenan-treated group, compared to the saline controls. Aδ-fibre-evoked responses of WDR neurones were significantly facilitated in carrageenan-treated rats (163 %) compared to saline-treated rats. There was a trend towards an increase in the input response in carrageenan-treated rats, but significance was not reached. There were no differences in the magnitudes of the other evoked responses of the WDR neurones in the carrageenan-treated group compared to saline controls.

**Table 1:** Comparison of characteristics of WDR neurones in carrageenan versus saline-treated rats

	Saline-treated (*n* = 15)	Carrageenan-treated (*n* = 38)	% Change vs saline-treated
Depth (μm)	677 ± 31	747 ± 21	NA
Threshold (mA)			
Aβ	0.14 ± 0.01	0.19 ± 0.01^##^	130
C	1.47 ± 0.08	1.81 ± 0.10*	124
Latency (ms)			
Aβ	7.6 ± 0.79	7.3 ± 0.40	96
C	182.3 ± 8.76	191.0 ± 8.50	105
Electrically evoked responses (number of APs)			
Aβ^a^	121 ± 10	126 ± 8	104
Aβ^b^	143 ± 10	145 ± 8	101
Aδ^b^	91 ± 16	148 ± 17^##^	163
C^b^	431 ± 49	497 ± 41	114
PD^b^	352 ± 49	381 ± 37	106
Input^b^	271 ± 51	368 ± 60	136
WU^b^	505 ± 60	502 ± 46	100

Characteristics of spinal WDR neurones recorded at approximately 30 h following intraplantar injection of 2 % λ-carrageenan or saline

*p < 0.05 unpaired *t* test, ^##^ *p* < 0.01 Mann-Whitney *U* test

*APs* action potentials, *NA* not applicable

^a^Responses electrically evoked by 3× Aβ-fibre threshold

^b^Responses electrically evoked by 3× C-fibre threshold

### Spinal AT-RvD1-inhibited responses of WDR neurones in the carrageenan model of hind paw inflammation

**Fig. 2:**
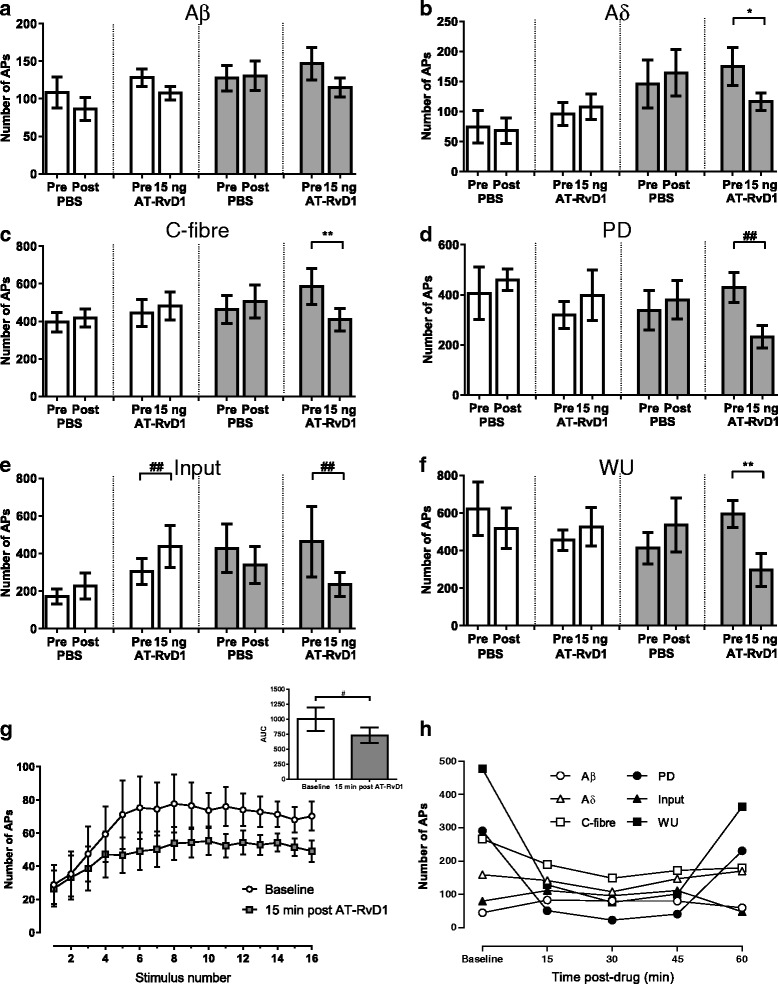
Effect of spinal administration of AT-RvD1 on evoked responses of WDR neurons. Spinal administration of AT-RvD1 selectively inhibited nociceptive fibre-evoked responses of spinal WDR neurones in carrageenan-treated rats, which were mediated mainly by FPR2/ALX receptor activation. In saline-treated rats (*white bars*), electrically evoked responses were not altered by PBS (*n* = 5) vehicle or AT-RvD1 (*n* = 10), except the input response which was slightly facilitated by AT-RvD1 (**e** *third and fourth bars*). AT-RvD1 (15 ng/50 μl) resulted in a non-significant decrease in Aβ-fibre-evoked responses (*p* = 0.0781) in carrageenan-treated rats (*grey bars*, *n* = 9). Spinal AT-RvD1 significantly suppressed WDR neurone responses evoked by electrical stimulation of Aδ and C fibres (**b**, **c**) as well as PD, input and WU **(d**–**f**) responses when compared to pre-drug responses. **g** Illustrates the effect of AT-RvD1 on WU of WDR neurones following 16 electrical stimulations in carrageenan-treated rats. At 15 min post AT-RvD1, the AUC was significantly lowered when compared to baseline (**g** *inset*). **h** Is an example of the electrically evoked responses of a WDR neurone recorded for 60 min following AT-RvD1 application in a carrageenan-treated rat. Data in **a**–**f** are mean maximal number of action potentials post drug application. *p < 0.05, **p < 0.01 paired *t* test. ^##^ *p* < 0.001 Wilcoxon test, *n* = 8–10 group, except for saline-PBS *n* = 5. *APs* action potentials

The mean time of spinal administration of AT-RvD1 was 29.2 ± 0.2 h (range 26.3–32.2 h) post carrageenan injection. Effects of AT-RvD1 versus PBS on evoked responses of spinal neurones were determined for 60 min after application to the surface of the spinal cord. Spinal administration of PBS did not alter any parameters in either saline- or carrageenan-treated rats. In the carrageenan-treated group, responses of 9 out of 10 neurones were inhibited by spinal administration of AT-RvD1. Maximal inhibition of evoked responses was observed between 25 and 39 min post treatment. Spinal administration of AT-RvD1 (15 ng) did not significantly alter Aβ-fibre-evoked responses in carrageenan-treated rats (Fig. 2a). This treatment did however significantly attenuate Aδ- and C-fibre-evoked responses WU and PD of WDR neurones in carrageenan-treated rats but not saline-treated rats (Fig. 2b–d, f and Table 2). There was a significant increase in input responses following treatment with spinal administration of AT-RvD1 in saline-treated rats (Fig. 2e). AUC of the stimulus-response curve representing WU following AT-RvD1 versus PBS was significantly different (Fig. 2g, *p* < 0.05). An example of evoked responses of a WDR neurone following AT-RvD1 administration in a carrageenan-treated rat is shown in Fig. 2h.

**Table 2:** Mean maximal percentage inhibition of evoked responses by AT-RvD1

Group effect (% maximal inhibition)			
	Saline-treated	Carrageenan-treated	
	AT-RvD1 (*n* = 10)	AT-RvD1 (*n* = 9)	AT-RvD1 post BOC-2 (*n* = 8)^a^
Aβ	2 ± 9	11 ± 13	5 ± 9
Aδ	−14 ± 12	27 ± 7*	−13 ± 8^#^
C	−10 ± 10	29 ± 5**	−1 ± 6^#^
PD	−19 ± 23	46 ± 8	−9 ± 14^#^
Input	−19 ± 15	41 ± 8*	−9 ± 14
WU	−10 ± 15	53 ± 12**	−1 ± 7^#^

Comparison of group effects of AT-RvD1 (15 ng/50 μl) on electrically evoked responses of spinal WDR neurones in carrageenan-treated rats versus saline-treated rats and the effects of pre-treatment with BOC-2 50 μg/50 μl (expressed as % maximal inhibition). AT-RvD1 significantly inhibited a number of evoked parameters in carrageenan-treated-rats but not saline-treated rats. Pre-treatment with BOC-2 significantly prevented the inhibitory effects of AT-RvD1 for most of the parameters. Note that negative values indicate facilitation

*p < 0.05, **p < 0.01 compared to AT-RvD1 in the saline-treated group, ^#^ *p* < 0.05 compared to AT-RvD1 in the carrageenan-treated group without BOC-2, Kruskal-Wallis test with Dunn’s post hoc test

^a^One neurone was inhibited by BOC-2 and was excluded from further analysis

To investigate the potential role of the FPR2/ALX receptor in the effects of AT-RvD1 on evoked responses of spinal neurones, the effects of spinal administration of BOC-2 (50 μg/50 μl) was tested in carrageenan-treated rats (*n* = 9 rats). Spinal administration of BOC-2 alone did not alter the evoked firing of spinal neurones compared to pre-drug (Additional file 1: Figure S2). A 15-min spinal pre-treatment with BOC-2 inhibited the effects of AT-RvD1 on evoked neuronal responses in carrageenan-treated rats (*n* = 8; Table 2). There were significant differences in the mean maximal percentage inhibition of evoked responses of WDR neurones by AT-RvD1 in the presence versus absence of BOC-2 in carrageenan-treated rats (Table 2).

### AT-RvD1 had minimal effect on WDR neurone responses in the MIA model of joint pain

Intra-articular injection of MIA resulted in significant weight bearing asymmetry, lowered hind paw withdrawal thresholds and joint pathology consistent with previous studies (Table 3). The baseline characteristics of spinal WDR neurones in MIA-treated rats were comparable to saline-treated rats (Table 3). At 28–32 days following model induction, rats were prepared for spinal recordings. Neurone depths, thresholds and latencies of Aβ and C-fibre of neurons in MIA-treated rats (*n* = 9) were comparable to those in saline-treated rats (*n* = 7).

**Table 3:** Comparison of pain behaviour, joint pathology and characteristics of WDR neurones in the MIA model of OA pain

	Saline-treated (*n* = 9)	MIA-treated (*n* = 11)	% change vs saline-treated
Pain behaviour day 28			
% weight bearing difference	2 ± 2	15 ± 2**	NA
PWT (g)	17 ± 2.7	6 ± 0.8**	NA
Knee joint pathology			
Macroscopic score	0 ± 0.14	14 ± 1.5****	NA
Presence of osteophytes	0/9	10/11	NA
Number of analysed neurones	7	9	
Depth (μm)	780 ± 48	720 ± 47	
Threshold (mA)			
Aβ	0.14 ± 0.01	0.14 ± 0.01	100
C	1.41 ± 0.12	1.54 ± 0.14	109
Latency (ms)			
Aβ	7.4 ± 1.04	7.5 ± 0.73	101
C	187.6 ± 24	141.1 ± 15	75
Electrically evoked responses (number of APs)			
Aβ^a^	129 ± 14	121 ± 14	94
Aβ^b^	151 ± 12	135 ± 12	89
Aδ^b^	146 ± 16	118 ± 19	81
C^b^	384 ± 52	419 ± 49	109
PD^b^	394 ± 42	365 ± 67	93
Input^b^	280 ± 52	362 ± 61	129
WU^b^	502 ± 63	426 ± 56	85

Pain behaviour (weight bearing difference and hind paw withdrawal thresholds) was assessed at 28 days following intra-articular injection of MIA. Spinal WDR neurones were recorded at 28–32 days following intra-articular injection of MIA or saline. Knee joint pathology was assessed following electrophysiological recordings

*APs* action potentials, *NA* not applicable for behavioural data

**p < 0.01, ****p < 0.0001 Mann-Whitney *U* test

^a^Responses electrically evoked by 3× Aβ-fibre threshold

^b^Responses electrically evoked by 3× C-fibre threshold

**Fig. 3:**
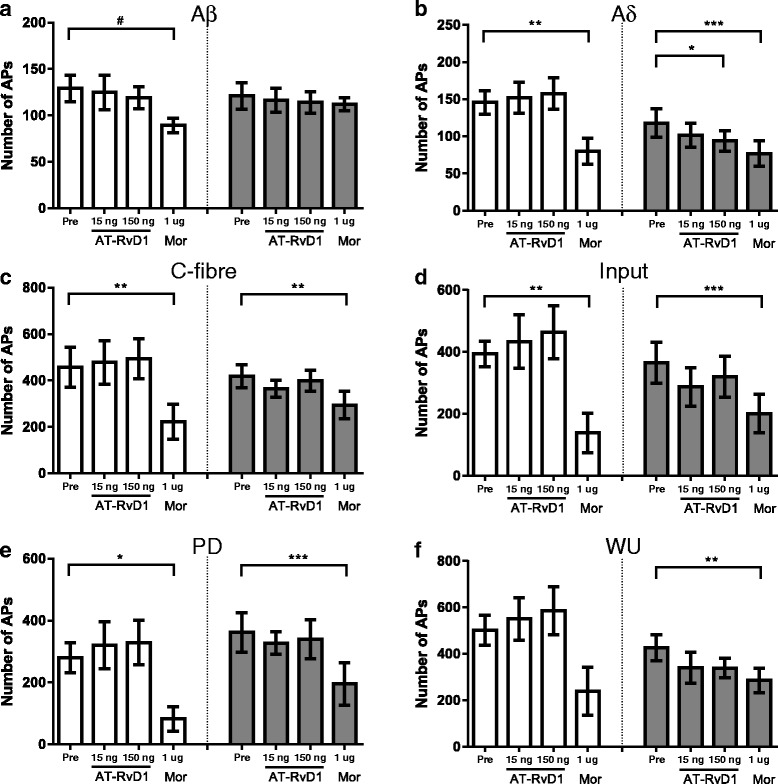
Minimal effects of AT-RvD1 on spinal WDR neurones in MIA-treated rats. On day 28 post model induction (intra-articular 1 mg MIA or saline), AT-RvD1 15 and 150 ng/50 μl applied spinally did not alter spinal WDR neurone firing in saline-treated rats (**a-f** *white bars*). In MIA-treated rats (**a-f** *grey bars*), even at the higher concentration, AT-RvD1 significantly inhibited only Aδ responses (**b**). However, WDR neurone firings in both groups were markedly suppressed following 1 μg/50 μl morphine (*Mor*) application. Data expressed as mean maximal number of action potentials post drug application. *p < 0.05, **p < 0.01, ***p < 0.0001 repeated measure ANOVA with Sidak post hoc test. ^#^ *p* < 0.05 Friedman test with Dunn’s post hoc test. Saline *n* = 7, MIA *n* = 9. Note that two neurones from each group were excluded due to incomplete data sets (not receiving morphine) and identified as outliers

In general, spinal administration of AT-RvD1 (15 and 150 ng) did not alter evoked responses of spinal neurones in either MIA- or saline-treated rats (Fig. 3a–f). To ascertain whether there was a shift in efficacy of treatment, a higher dose of AT-RvD1 (150 ng) was also studied. This dose produced a small but significant inhibition of Aδ-fibre-evoked responses of WDR neurones in MIA-treated rats, compared to baseline (Fig. 3b), but did not alter other responses. Spinal administration of morphine (1 μg/50 μl) significantly attenuated Aδ- and C-fibre-evoked responses of spinal neurones in both MIA- and saline-treated rats (Fig. 3a–f). Morphine also produced a small but significant inhibition of Aβ-fibre-evoked responses of WDR neurones in saline-treated rats (Fig. 3a). Comparison of the maximal effect of morphine on evoked responses of WDR neurones revealed no significant differences between MIA- and saline-treated rats (Table 4).

**Table 4:** Effects of spinal AT-RvD1 versus morphine on evoked responses of spinal neurones in MIA- and saline-treated rats

	Saline-treated (*n* = 7)			MIA-treated (*n* = 9)		
	AT-RvD1		Morphine 1 μg	AT-RvD1		Morphine 1 μg
	15 ng	150 ng		15 ng	150 ng	
Aβ	5 ± 7	6 ± 5	28 ± 6	1 ± 8	1 ± 9	2 ± 7^#^
Aδ	−5 ± 10	−10 ± 13	47 ± 7	11 ± 8	14 ± 10	40 ± 6
C	−4 ± 7	−13 ± 9	60 ± 14	10 ± 7	3 ± 8	35 ± 8
PD	−6 ± 14	−16 ± 15	70 ± 12	16 ± 24	9 ± 14	55 ± 9
Input	−5 ± 15	−19 ± 21	71 ± 12	−2 ± 12	2 ± 14	58 ± 11
WU	−12 ± 13	−20 ± 16	62 ± 13	17 ± 19	12 ± 12	34 ± 6

Comparison of group effects of AT-RvD1 (15, 150 ng/50 μl) and morphine (1 μg/50 μl) on electrically evoked responses of spinal WDR neurones in MIA-treated rats versus saline-treated rats, expressed as % maximal inhibition. Note that negative values indicate facilitation

^#^ *p* < 0.05 Mann-Whitney *U* test versus saline-treated rats

### The impact of peripheral inflammation on the spinal expression of the resolvin receptors and related enzymatic pathways

**Fig. 4:**
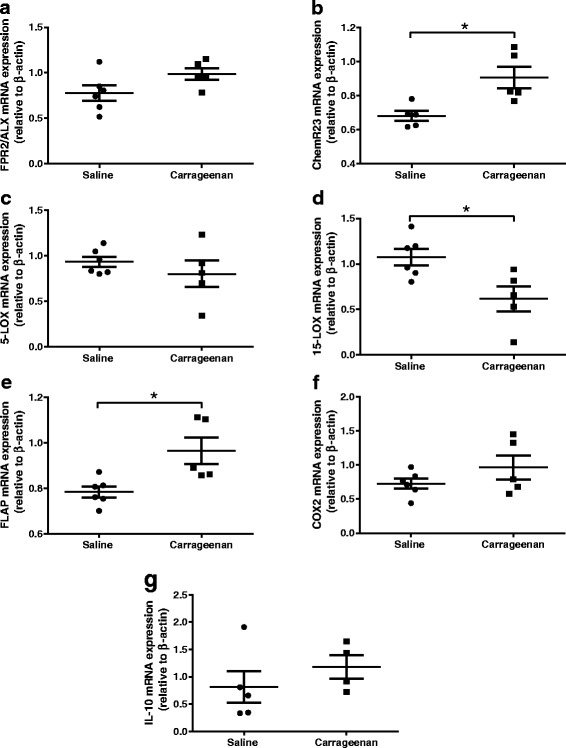
Changes in gene expressions in spinal cord post intraplantar injection of saline or carrageenan. The ipsilateral dorsal horns of spinal cords were collected at 30 h post injection; mRNA levels are relative to β-actin. mRNA levels of receptors for resolvin D and resolvin E—FPR2/ALX (**a**) and ChemR23 (**b**), respectively, enzymes involved in resolvin biosynthesis-5-LOX (**c**) and 15-LOX (**d**), activating protein for 5-LOX—FLAP (**e**), pro-inflammatory enzyme—COX-2 (**f**) and anti-inflammatory cytokine—IL-10 (**g**). Data expressed as mean ± SEM. *p < 0.05 Mann-Whitney *U* test. Saline-treated *n* = 6, carrageenan-treated *n* = 5. *FPR2/ALX* formyl peptide receptor 2, *ChemR23* chemerin receptor 23, *FLAP* 5-lipoxygenase-activating protein, *LOX* lipoxygenase, *COX-2* cyclooxygenase-2, *IL-10* interleukin-10

In separate groups of rats, ipsilateral dorsal horns of the spinal cord were collected at 30 h following intraplantar injection of carrageenan. β-actin messenger RNA (mRNA) expression was comparable in both groups of rats (saline 0.3068 ± 0.013 versus carrageenan 0.2965 ± 0.016) serving as a suitable reference gene for relative comparisons. Following intraplantar injection of carrageenan, levels of FPR2/ALX mRNA remained stable in the dorsal horn of the spinal cord, compared to saline-treated rats (Fig. 4a). Carrageenan treatment was associated with a significant increase (1.5-fold, *p* < 0.05) in ChemR23 mRNA expression (Fig. 4b). Focusing on enzymes involved in resolvin biosynthesis, 5-LOX mRNA expression in the dorsal horn of the spinal cord was similar in carrageenan- and saline-treated rats (Fig. 4c). However, expression of the 5-LOX-activating protein FLAP was significantly higher (1.3-fold increase, *p* < 0.05) in the dorsal horn of the spinal cord of carrageenan-treated rats, compared to saline controls (Fig. 4e). Expression of 15-LOX mRNA level was significantly lower in carrageenan-treated rats when compare to saline-treated rats (*p* < 0.05, Fig. 4d).

## Discussion

Previous studies reported acute inhibitory effects of spinal resolvins on behavioural responses to painful stimuli. The results reported herein demonstrate that spinal administration of AT-RvD1 did not alter physiological spinal nociceptive responses but significantly attenuated evoked nociceptive responses of dorsal horn spinal neurones following a 24-h period of peripheral inflammation of the hind paw. At the timepoint of the novel effects of spinal AT-RvD1, we demonstrate that the model of hind paw inflammation is associated with changes in the dorsal horn gene expression of enzymes related to resolvin pathways. The novel inhibitory effect of spinal AT-RvD1 was not recapitulated in a model of chronic joint arthritis, suggesting specificity to conditions associated with overt peripheral inflammation.

The carrageenan model was associated with robust pain behaviour, altered weight bearing and decreased PWT, consistent with previous studies at early timepoints [20, 35]. In the present study, we extended the period following carrageenan injection to allow the characterisation of spinal events at timepoints more relevant to injuries associated with acute inflammation and pain seen clinically and the timecourse of the activation of the endogenous resolution pathways [36]. At the timepoint of spinal neuronal recordings, Aδ-evoked responses of WDR neurones significantly increased compared to the control group. All other evoked responses of WDR neurones were similar in saline- and carrageenan-treated rats. Clearly, a limitation of quantifying the behavioural pain response and recording the responses of WDR neurones at this later timepoint was that baseline responses of neurones pre-carrageenan injection could not be characterised, and therefore, only population changes in response can be reported. Previously, WDR neuronal responses characterised before and at 3 h post carrageenan injection were reported to exhibit increased C-fibre-evoked responses in around half the group, and the rest had decreased response [26]. In our study, 22 (58 %) neurons in carrageenan rats displayed higher baseline C-fibre responses compared to the mean of baseline C-fibre responses in non-inflamed animals, similar to previously reported [26].

Spinal administration of AT-RvD1 attenuated C-fibre-evoked responses of spinal neurones in carrageenan-treated rats but not in saline controls. The magnitude of the inhibitory effects of AT-RvD1 for both non-facilitated (input) and facilitated (post-discharge) C-fibre responses of WDR neurones were comparable. WU was the most sensitive to the effects of AT-RvD1 (53 % inhibited). These data provide for the first time a neurophysiological basis for the effects of a resolvin molecule on spinal nociception in vivo and reveal that these inhibitory effects are only evident under certain conditions. Our results are in agreement with other ex vivo spinal cord slice studies which demonstrated that RvE1 selectively reduces excitatory post-synaptic potential in the presence of the pro-inflammatory cytokine tumour necrosis factor-α (TNF-α), by inhibiting *N*-methyl-d-aspartate (NMDA) receptor activation [14], and RvD2 selectively attenuates long-term potentiation of spinal neurones from inflamed animals [37]. To date, the reported analgesic and anti-inflammatory effects of resolvins in animal pain models [13–15, 17] were hypothesised to be mediated by both peripheral and central nervous system sites of action [14]. Our demonstration of a direct spinal effect on neuronal responses in vivo provides a neuronal basis for the behavioural evidence that spinal AT-RvD1 reduces carrageenan-induced mechanical hypersensitivity at earlier timepoints [13]. Timecourse analysis of the effects of AT-RvD1 revealed peak effects 30 min post administration, with responses returning to control levels within the hour (as shown in Additional file 1: Figure S3). Although AT-RvD1 is more resistant to metabolism compared to RvD1 [9], the short-lived nature of action of these bioactive lipids is a disadvantage that needs to be overcome if they are to be harnessed as analgesics [10, 38]. The inhibitory effects of AT-RvD1 on evoked responses of WDR neurones were blocked by BOC-2, an antagonist for the receptor FPR2/ALX which is thought to be coupled to Gi [11] and in principle capable of reducing neuronal excitability. Spinal administration of BOC-2 alone did not alter evoked neuronal responses in inflamed rats, suggesting limited tonic inhibition of spinal neuronal activity.

Spinal AT-RvD1 did not inhibit nociceptive responses of WDR neurones in the absence of an earlier overt inflammatory stimulus (saline-treated) at the doses studied. Although it is possible that higher spinal doses of AT-RvD1 may alter physiological spinal nociception in control rats, the greater effectiveness of spinal AT-RvD1 following peripheral inflammation suggests that treatments targeting this mechanism may have a window of selectivity for inflammatory pain.

The MIA model of arthritis pain is rapidly developing (1–2 weeks post induction) and associated with knee joint features characteristic of human osteoarthritis and pain behaviour [21, 22, 34, 39]. In the present study, the effects of spinal AT-RvD1 on evoked responses of spinal neurones were assessed at 28 days following intra-articular injection of MIA pain, a timepoint when spinal neuronal responses and weight bearing asymmetry are correlated [21] and there is an increase in spinal glial fibrillary acidic protein (GFAP) immunofluorescence, a marker of spinal astrocyte sensitization. Reduction in PWT distal to the site of pathology in the MIA model is considered to represent centrally mediated receptive field expansion in osteoarthritis (OA) [31, 40]. Thus, delivery of the electrical stimulation at the hind paw was used to investigate the effect of AT-RvD1 on central sensitisation in the MIA model. Spinal administration of AT-RvD1, at the higher dose, produced a modest inhibition of Aδ-fibre responses in MIA-treated rats, but all other evoked responses of WDR neurones were unaltered. However, our positive control spinal morphine given at the end of the experiment clearly inhibited the evoked responses of spinal neurones recorded in MIA rats.

The differential effect of AT-RvD1 on spinal nociceptive transmission following a period of overt peripheral inflammation is suggestive of changes in the resolvin system, compared to control conditions. Spinal expression of two well-characterised resolvin receptors FPR2/ALX and ChemR23 was quantified in the dorsal horn of the spinal cord in carrageenan- and saline-treated rats. FPR2/ALX is activated by RvD1, and AT-RvD1 and is predominantly localised with GFAP [13, 30]. ChemR23 is expressed by neurones [14] and localised with substance P in the central terminals of primary afferents in the superficial dorsal horn [14]. There was no change in FPR2/ALX receptor mRNA level at 24 h post carrageenan injection, compared to controls. There was however a significant elevation in spinal expression of ChemR23 mRNA in the carrageenan model of inflammatory pain compared to controls (Fig. 4b). This observation is consistent with the report that ChemR23 gene expression is increased in chronic constriction model of neuropathic pain, but not in CFA-induced paw inflammation, at days 3 and 14 post model induction [41]. Our observation that spinal expression of ChemR23 is increased, while FPR2/ALX was unaltered, in the carrageenan model does not necessarily account for the increased effect of AT-RvD1 in this group of rats. Previous studies have reported that RvD1 (structurally very similar to AT-RvD1 [9]) has negligible effect at ChemR23, whereas resolvin E1 (RvE1) has an EC_50_ ∼1.3 × 10^−11^ M for ChemR23 [42]. On this basis, we suggest that it is unlikely that the increased effectiveness of AT-RvD1 arises as a result of an increased expression of ChemR23 in the spinal dorsal horn in the carrageenan model of inflammation.

In parallel with these studies, we also sought evidence for potential changes in the spinal gene expression of enzymes involved with resolvin biosynthesis following the period of hind paw inflammation. We report a significant decrease in the gene expression of 15-LOX, the first step enzyme converting DHA to 17S-hydroperoxy DHA (17S-H(p)-DHA) [43], in the dorsal horn of the spinal cord in the model of carrageenan inflammation. Expression of LOX-5, the final enzyme in the biosynthesis of RvD1 and RvE1, was however unaltered in the carrageenan model. Concomitantly, there was a significant increase in the expression of FLAP mRNA, a key enzyme mediating the generation of RvD1, in the dorsal horn of the spinal cord in carrageenan-treated rats compared to controls. Resolvins are endogenously generated during the resolution phase of inflammation [7], which is consistent with the timing of our electrophysiological and gene expression studies. Although not proven, increased expression of 15-LOX and FLAP in the dorsal horn of the spinal cord is likely to increase baseline synthesis of endogenous resolvins, which may directly enhance the inhibitory effects of exogenous AT-RvD1, or reduce the rate of catabolism of exogenous AT-RvD1, leading to an increased inhibitory effect on evoked neuronal responses. Interrogation of this proposal is not readily achievable as spinal blockade of LOXs and COX-2 has analgesic effects [44, 45] or inhibits neuronal firing [46]. Collectively, these data provide new evidence for complex changes in key enzymes involved in the biosynthesis of the resolvins in the spinal cord under specific conditions, which require further interrogation.

## Conclusions

In conclusion, spinal administration of AT-RvD1 only reduced peripheral nociceptive fibre-evoked responses of spinal WDR neurones in vivo in the presence of peripheral hind paw inflammation, with minimal effects on physiological spinal nociception. The ability of AT-RvD1 to selectively target inflammatory-driven spinal hyperexcitability of nociceptive pathways was associated with pathway-specific changes in the gene expression of enzymes known to mediate the generation of the resolvins. Our data support the further investigation of the analgesic potential of this class of molecules.

## Additional file

Additional file 1: Table S1. Group sizes of animals in the studies. **Table S2.** Sequences of primers and probes used in the gene expression study. **Figure S1.** Diagram illustrating resolvin biosynthetic pathways of resolvin D1 (RvD1 or 17S-RvD1), AT-RvD1 (17R-RvD1) and RvE1 (18R-RvE1) and their receptors. Genes of interest in the present study are indicated in red. Note that AT-RvD1 in the studies was exogenously administered. Abbreviations: ASA acetyl salicylic acid or aspirin, ChemR23 chemerin receptor, COX-2 cyclooxygenase, CYP450 cytochrome P450, DHA docosahexaenoic acid, FPR2/ALX formyl peptide receptor 2, 17R-H(p)-DHA 17-R-hydroperoxy docosahexaenoic acid, 17S-H(p)-DHA 17S-hydroperoxy docosahexaenoic acid, 15-LOX 15-lipoxygenase, 5-LOX 5-lipoxygenase. **Figure S2.** BOC-2 alone had no effect on spinal WDR neurone responses in carrageenan-treated rats. Spinal administration of BOC-2 50 μg/50 μl alone (*n* = 9) did not alter electrically evoked firing of WDR neurones in carrageenan-treated rats when compared to pre-drug responses (pre). **Figure S3.** Timecourse of the effects of AT-RvD1 on electrically evoked responses of spinal WDR neurones in carrageenan-treated rats. AT-RvD1 15 ng/50 μl was directly applied onto the exposed spinal cord after stable baseline responses were established. The inhibitory effects on C-fibre and post-discharge (PD) responses peaked at 15 min post application and returned towards control levels at 60 min. Aβ-fibre responses remained comparable to the control level throughout the hour (*n* = 9 neurones). **Figure S4.** An explanation of input and wind-up (WU). The graph illustrates responses in C-fibre and post-discharge bands (90–800 post stimulus) of a WDR neurone following a train of 16 electrical stimulations (0.5 Hz, 2-ms pulse width at 3× C-fibre threshold). If there is no potentiation, the responses will be flat, shown as the orange theoretical line. The input is calculated by taking the initial response (10) multiplied by the stimulus number (16) which results in 160. The input represents initial or non-potentiated response of neurones. However, in the actual experiment, a WDR neurone can display an increase in responsiveness after repetitive stimulation (shown as red line) which is a typical characteristic. This phenomenon is called wind-up (WU) which represents enhanced excitability. The cumulative number of action potentials evoked by the train of stimulation (490) minus input (160) results in calculated WU 330.

## Abbreviations

15-LOX: 15-Lipoxygenase
15-PGDH: 15-Hydroxyprostaglandin dehydrogenase
17R-RvD1: 17R-Resolvin D1
17S-H(p)-DHA: 17S-Hydroperoxy docosahexaenoic acid
17S-RvD1: 17S-Resolvin D1
3×: Three times
5-LOX: 5-Lipoxygenase
ANOVA: Analysis of variance
AP: Action potential
AT-RvD1: Aspirin-triggered resolvin D1
BOC-2: Butoxy carbonyl-Phe-Leu-Phe-Leu-Phe
cDNA: Complementary deoxyribonucleic acid
ChemR23: Chemerin receptor
COX-2: Cyclooxygenase-2
DHA: Docosahexaenoic acid
FLAP: 5-Lipoxygenase-activating protein
FPR2/ALX: Formyl peptide receptor 2
g: Gram
GFAP: Glial fibrillary acidic protein
GPR32: G-protein-coupled receptor 32
h: Hour
Hz: Hertz
IL-10: Interleukin-10
l: Litre
M: Molar
MIA: Monosodium iodoacetate
min: Minute
μl: Microlitre
μm: Micrometre
mm: Millimetre
ms: Millisecond
NeuN: Neuronal nuclear antigen
ng: Nanogram
NMDA: *N*-Methyl-d-aspartate
OA: Osteoarthritis
°C: Degree Celsius
PBS: Phosphate-buffered saline
PD: Post-discharge
PWT: Paw withdrawal threshold
RNA: Ribonucleic acid
RvD1: Resolvin D1
RvE1: Resolvin E1
SEM: Standard error of mean
SPMs: Specialised proresolving mediators
TNF-α: Tumour necrosis factor-α
WDR: Wide dynamic range
wt: Weight
WU: Wind-up

## References

[1] Grace PM, Hutchinson MR, Maier SF, Watkins LR. Pathological pain and the neuroimmune interface. Nat Rev Immunol 2014;14:217–31. 10.1038/nri3621 PMC5525062 24577438

[2] Luo C, Kuner T, Kuner R (2014). Synaptic plasticity in pathological pain. Trends Neurosci.

[3] Svensson CI, Marsala M, Westerlund A, Calcutt NA, Campana WM, Freshwater JD, Catalano R, Feng Y, Protter AA, Scott B, Yaksh TL (2003). Activation of p38 mitogen-activated protein kinase in spinal microglia is a critical link in inflammation-induced spinal pain processing. J Neurochem.

[4] Ji RR, Befort K, Brenner GJ, Woolf CJ (2002). ERK MAP kinase activation in superficial spinal cord neurons induces prodynorphin and NK-1 upregulation and contributes to persistent inflammatory pain hypersensitivity. J Neurosci.

[5] Okine BN, Norris LM, Woodhams S, Burston J, Patel A, Alexander SP, Barrett DA, Kendall DA, Bennett AJ, Chapman V (2012). Lack of effect of chronic pre-treatment with the FAAH inhibitor URB597 on inflammatory pain behaviour: evidence for plastic changes in the endocannabinoid system. Br J Pharmacol.

[6] Recchiuti A, Serhan CN (2012). Pro-resolving lipid mediators (SPMs) and their actions in regulating miRNA in novel resolution circuits in inflammation. Front Immunol.

[7] Serhan CN (2014). Pro-resolving lipid mediators are leads for resolution physiology. Nature.

[8] Serhan CN, Hong S, Gronert K, Colgan SP, Devchand PR, Mirick G, Moussignac RL (2002). Resolvins: a family of bioactive products of omega-3 fatty acid transformation circuits initiated by aspirin treatment that counter proinflammation signals. J Exp Med.

[9] Sun YP, Oh SF, Uddin J, Yang R, Gotlinger K, Campbell E, Colgan SP, Petasis NA, Serhan CN (2007). Resolvin D1 and its aspirin-triggered 17R epimer. Stereochemical assignments, anti-inflammatory properties, and enzymatic inactivation. J Biol Chem.

[10] Orr SK, Colas RA, Dalli J, Chiang N, Serhan CN (2015). Proresolving actions of a new resolvin D1 analog mimetic qualifies as an immunoresolvent. Am J Physiol Lung Cell Mol Physiol.

[11] Chiang N, Serhan CN, Dahlen SE, Drazen JM, Hay DW, Rovati GE, Shimizu T, Yokomizo T, Brink C (2006). The lipoxin receptor ALX: potent ligand-specific and stereoselective actions in vivo. Pharmacol Rev.

[12] Back M, Powell WS, Dahlen SE, Drazen JM, Evans JF, Serhan CN, Shimizu T, Yokomizo T, Rovati GE (2014). Update on leukotriene, lipoxin and oxoeicosanoid receptors: IUPHAR Review 7. Br J Pharmacol.

[13] Abdelmoaty S, Wigerblad G, Bas DB, Codeluppi S, Fernandez-Zafra T, El-Awady el S, Moustafa Y, Abdelhamid Ael D, Brodin E, Svensson CI (2013). Spinal actions of lipoxin A4 and 17(R)-resolvin D1 attenuate inflammation-induced mechanical hypersensitivity and spinal TNF release. PLoS One.

[14] Xu ZZ, Zhang L, Liu T, Park JY, Berta T, Yang R, Serhan CN, Ji RR (2010). Resolvins RvE1 and RvD1 attenuate inflammatory pain via central and peripheral actions. Nat Med.

[15] Xu ZZ, Berta T, Ji RR (2013). Resolvin E1 inhibits neuropathic pain and spinal cord microglial activation following peripheral nerve injury. J Neuroimmune Pharmacol.

[16] Huang L, Wang CF, Serhan CN, Strichartz G (2011). Enduring prevention and transient reduction of postoperative pain by intrathecal resolvin D1. Pain.

[17] Lima-Garcia JF, Dutra RC, da Silva K, Motta EM, Campos MM, Calixto JB (2011). The precursor of resolvin D series and aspirin-triggered resolvin D1 display anti-hyperalgesic properties in adjuvant-induced arthritis in rats. Br J Pharmacol.

[18] Zimmermann M (1983). Ethical guidelines for investigations of experimental pain in conscious animals. Pain.

[19] Kilkenny C, Browne WJ, Cuthill IC, Emerson M, Altman DG (2012). Improving bioscience research reporting: the ARRIVE guidelines for reporting animal research. Osteoarthritis Cartilage.

[20] Jhaveri MD, Richardson D, Robinson I, Garle MJ, Patel A, Sun Y, Sagar DR, Bennett AJ, Alexander SP, Kendall DA (2008). Inhibition of fatty acid amide hydrolase and cyclooxygenase-2 increases levels of endocannabinoid related molecules and produces analgesia via peroxisome proliferator-activated receptor-alpha in a model of inflammatory pain. Neuropharmacology.

[21] Sagar DR, Staniaszek LE, Okine BN, Woodhams S, Norris LM, Pearson RG, Garle MJ, Alexander SP, Bennett AJ, Barrett DA (2010). Tonic modulation of spinal hyperexcitability by the endocannabinoid receptor system in a rat model of osteoarthritis pain. Arthritis Rheum.

[22] Sagar DR, Nwosu L, Walsh DA, Chapman V (2015). Dissecting the contribution of knee joint NGF to spinal nociceptive sensitization in a model of OA pain in the rat. Osteoarthritis Cartilage.

[23] Dixon WJ (1980). Efficient analysis of experimental observations. Annu Rev Pharmacol Toxicol.

[24] Chaplan SR, Bach FW, Pogrel JW, Chung JM, Yaksh TL (1994). Quantitative assessment of tactile allodynia in the rat paw. J Neurosci Methods.

[25] Chapman V, Suzuki R, Dickenson AH (1998). Electrophysiological characterization of spinal neuronal response properties in anaesthetized rats after ligation of spinal nerves L5-L6. J Physiol.

[26] Stanfa LC, Sullivan AF, Dickenson AH (1992). Alterations in neuronal excitability and the potency of spinal mu, delta and kappa opioids after carrageenan-induced inflammation. Pain.

[27] Stanfa LC, Dickenson AH (2004). In vivo electrophysiology of dorsal-horn neurons. Methods Mol Med.

[28] Mendell LM (1966). Physiological properties of unmyelinated fiber projection to the spinal cord. Exp Neurol.

[29] Stenfeldt AL, Karlsson J, Wenneras C, Bylund J, Fu H, Dahlgren C (2007). Cyclosporin H, Boc-MLF and Boc-FLFLF are antagonists that preferentially inhibit activity triggered through the formyl peptide receptor. Inflammation.

[30] Wang ZF, Li Q, Liu SB, Mi WL, Hu S, Zhao J, Tian Y, Mao-Ying QL, Jiang JW, Ma HJ (2014). Aspirin-triggered Lipoxin A4 attenuates mechanical allodynia in association with inhibiting spinal JAK2/STAT3 signaling in neuropathic pain in rats. Neuroscience.

[31] Sagar DR, Burston JJ, Hathway GJ, Woodhams SG, Pearson RG, Bennett AJ, Kendall DA, Scammell BE, Chapman V (2011). The contribution of spinal glial cells to chronic pain behaviour in the monosodium iodoacetate model of osteoarthritic pain. Mol Pain.

[32] Erhuma A, Salter AM, Sculley DV, Langley-Evans SC, Bennett AJ (2007). Prenatal exposure to a low-protein diet programs disordered regulation of lipid metabolism in the aging rat. Am J Physiol Endocrinol Metab.

[33] Morris CJ (2003). Carrageenan-induced paw edema in the rat and mouse. Methods Mol Biol.

[34] Guingamp C, Gegout-Pottie P, Philippe L, Terlain B, Netter P, Gillet P (1997). Mono-iodoacetate-induced experimental osteoarthritis: a dose-response study of loss of mobility, morphology, and biochemistry. Arthritis Rheum.

[35] Hargreaves K, Dubner R, Brown F, Flores C, Joris J (1988). A new and sensitive method for measuring thermal nociception in cutaneous hyperalgesia. Pain.

[36] Bannenberg GL, Chiang N, Ariel A, Arita M, Tjonahen E, Gotlinger KH, Hong S, Serhan CN (2005). Molecular circuits of resolution: formation and actions of resolvins and protectins. J Immunol.

[37] Park CK, Xu ZZ, Liu T, Lu N, Serhan CN, Ji RR (2011). Resolvin D2 is a potent endogenous inhibitor for transient receptor potential subtype V1/A1, inflammatory pain, and spinal cord synaptic plasticity in mice: distinct roles of resolvin D1, D2, and E1. J Neurosci.

[38] Arita M, Oh SF, Chonan T, Hong S, Elangovan S, Sun YP, Uddin J, Petasis NA, Serhan CN (2006). Metabolic inactivation of resolvin E1 and stabilization of its anti-inflammatory actions. J Biol Chem.

[39] Pomonis JD, Boulet JM, Gottshall SL, Phillips S, Sellers R, Bunton T, Walker K (2005). Development and pharmacological characterization of a rat model of osteoarthritis pain. Pain.

[40] Arendt-Nielsen L, Nie H, Laursen MB, Laursen BS, Madeleine P, Simonsen OH, Graven-Nielsen T (2010). Sensitization in patients with painful knee osteoarthritis. Pain.

[41] Rodriguez Parkitna J, Korostynski M, Kaminska-Chowaniec D, Obara I, Mika J, Przewlocka B, Przewlocki R (2006). Comparison of gene expression profiles in neuropathic and inflammatory pain. J Physiol Pharmacol.

[42] Krishnamoorthy S, Recchiuti A, Chiang N, Yacoubian S, Lee CH, Yang R, Petasis NA, Serhan CN (2010). Resolvin D1 binds human phagocytes with evidence for proresolving receptors. Proc Natl Acad Sci U S A.

[43] Serhan CN, Petasis NA (2011). Resolvins and protectins in inflammation resolution. Chem Rev.

[44] Trang T, McNaull B, Quirion R, Jhamandas K (2004). Involvement of spinal lipoxygenase metabolites in hyperalgesia and opioid tolerance. Eur J Pharmacol.

[45] Gregus AM, Doolen S, Dumlao DS, Buczynski MW, Takasusuki T, Fitzsimmons BL, Hua XY, Taylor BK, Dennis EA, Yaksh TL (2012). Spinal 12-lipoxygenase-derived hepoxilin A3 contributes to inflammatory hyperalgesia via activation of TRPV1 and TRPA1 receptors. Proc Natl Acad Sci U S A.

[46] Staniaszek LE, Norris LM, Kendall DA, Barrett DA, Chapman V (2010). Effects of COX-2 inhibition on spinal nociception: the role of endocannabinoids. Br J Pharmacol.

