# Supplementary material for: Inhibitory effects of aspirin-triggered resolvin D1 on spinal nociceptive processing in rat pain models
Source: J Neuroinflammation. 2016 Sep 2;13(1):233. doi: 10.1186/s12974-016-0676-6 (PMC5010669; doi:10.1186/s12974-016-0676-6)
Supplement: Additional file 1: Table S1. — Group sizes of animals in the studies. Table S2. Sequences of primers and probes used in the gene expression study. Figure S1. Diagram illustrating resolvin biosynthetic pathways of resolvin D1 (RvD1 or 17S-RvD1), AT-RvD1 (17R-RvD1) and RvE1 (18R-RvE1) and their receptors. Genes of interest in the present study are indicated in red. Note that AT-RvD1 in the studies was exogenously administered. Abbreviations: ASA acetyl salicylic acid or aspirin, ChemR23 chemerin receptor, COX-2 cyclooxygenase, CYP450 cytochrome P450, DHA docosahexaenoic acid, FPR2/ALX formyl peptide receptor 2, 17R-H(p)-DHA 17-R-hydroperoxy docosahexaenoic acid, 17S-H(p)-DHA 17S-hydroperoxy docosahexaenoic acid, 15-LOX 15-lipoxygenase, 5-LOX 5-lipoxygenase. Figure S2. BOC-2 alone had no effect on spinal WDR neurone responses in carrageenan-treated rats. Spinal administration of BOC-2 50 μg/50 μl alone (n = 9) did not alter electrically evoked firing of WDR neurones in carrageenan-treated rats when compared to pre-drug responses (pre). Figure S3. Timecourse of the effects of AT-RvD1 on electrically evoked responses of spinal WDR neurones in carrageenan-treated rats. AT-RvD1 15 ng/50 μl was directly applied onto the exposed spinal cord after stable baseline responses were established. The inhibitory effects on C-fibre and post-discharge (PD) responses peaked at 15 min post application and returned towards control levels at 60 min. Aβ-fibre responses remained comparable to the control level throughout the hour (n = 9 neurones). Figure S4. An explanation of input and wind-up (WU). The graph illustrates responses in C-fibre and post-discharge bands (90–800 post stimulus) of a WDR neurone following a train of 16 electrical stimulations (0.5 Hz, 2-ms pulse width at 3× C-fibre threshold). If there is no potentiation, the responses will be flat, shown as the orange theoretical line. The input is calculated by taking the initial response (10) multiplied by the stimulus number (16) which results in 160. The i [file 12974_2016_676_MOESM1_ESM.docx]

**Supplementary materials**

**Table S1 Group sizes of animals for in the studies**

| **Method** | **Model** | **Spinal treatment** | **Group Size** | **No. of neurones analysed** | **Exclusions** |
| --- | --- | --- | --- | --- | --- |
| Electro-physiology | Intraplantar saline | PBS | 5 | 5 |  |
|  | Intraplantar saline | AT-RvD1 | 10 | 10 |  |
|  | Intraplantar carrageenan | PBS | 10 | 10 |  |
|  | Intraplantar carrageenan | AT-RvD1 | 10 | 9 | Excluded 1 neurone - facilitated |
|  | Intraplantar carrageenan | BOC-2 | 9 | 9 |  |
|  | Intraplantar carrageenan | BOC-2-AT-RvD1 | 9 | 8 | Excluded 1 neurone -inhibited |
|  |  | Total | 53 |  |  |
|  | Intra-articular saline | AT-RvD1-Morphine | 9 | 7 | Excluded 1 neurone outlier and  1 neurone incomplete data set (no morphine) |
|  | Intra-articular MIA | AT-RvD1-Morphine | 11 | 9 | Excluded 1 neurone outlier and  1 neurone incomplete data set (no morphine) |
|  |  | Total | 20 |  |  |
| Gene expression | Intraplantar saline | N/A | 6 | N/A |  |
|  | Intraplantar carrageenan | N/A | 5 | N/A |  |
|  |  | Total | 11 | N/A |  |
|  |  | Total | 84 | 67 |  |

**Table S2 Sequences of primers and probes used in gene expression study**

| **Gene** | **Forward primer** | **Reverse primer** | **Taqman probe** |
| --- | --- | --- | --- |
| **Beta actin** | AGGCCATGTACGTAGCCATCCA | TCTCCGGAGTCCATCACAATG | TGTCCCTGTATGCCTCTGGTCGTACCAC |
| **ALX** | CTTGGACCGCTGCATTTGT | CCTTCCTAGCCAGGCTCACA | CAGTCTGGGCTCAGAACCACCGC |
| **ChemR23*** | AGGACCTACCCTCGAGTTCTATTCT | CGTAGATGCTGGAGTCGTTGTAA | TCCAAAGAGATGGAGTACGA |
| **COX-2** | GGCACAAATATGATGTTCGCA | CTCGCTTCTGATCTGTCTTGA | TCTTTGCCCAGCACTTCACTCATCAGTTT |
| **FLAP** | CCCCACTTTCCTTGTGGTACTC | TGCCTCACGAACAGATACATCAG | AGCCAAGTCCCCGCCGCCT |
| **IL-10** | GAAGCTGAAGACCCTCTGGATACA | CCTTTGTCTTGGAGCTTATTAAAATCA | CGCTGTCATCGATTTCTCCCCTGTGA |
| **5-LOX*** | TGGTGTCTGAGGTGTTCGGTAT | GGCAATGGTGAACCTCACATG | CCCTTTTCAAGCTGCTG |
| **15-LOX** | TGATGCCTGATGGACAACTCTT | CCG AGG GCG TGA AAA TAG G | CCATAGCCATCCAGCTTGAACTTC CCA |

*conjugated minor groove binder (MGB) probe

**Figure S1 Diagram illustrating resolvin biosynthetic pathways of resolvin D1 (RvD1 or 17S-RvD1), AT-RvD1 (17R-RvD1) and RvE1 (18R-RvE1) and their receptors.** Genes of interest in the present study are indicated in red. Note that AT-RvD1 in the studies was exogenously administered. Abbreviations; ASA: acetyl salicylic acid or aspirin, ChemR23: chemerin receptor, COX-2:cyclooxygenase, CYP450:cytochrome P450, DHA: docosahexaenoic acid, FPR2/ALX: formylpeptide receptor 2, 17R-H(p)-DHA:17-R-hydroperoxy docosahexaenoic acid, 17S-H(p)-DHA:17S-hydroperoxy docosahexaenoic acid, 15-LOX:15-lipoxegenase, 5-LOX:5-lipoxegense.

**Figure S2 BOC-2 alone had no effect on spinal WDR neurone responses in carrageenan-treated rats**. Spinal administration of BOC-2 50 μg 50/μl alone (n=9) did not alter electrically evoked firing of WDR neurones in carrageenan-treated rats when compared to pre-drug responses (pre).

**Figure S3** **Timecourse of the effects of AT-RvD1 on electrically evoked responses of spinal WDR neurones in carrageenan-treated rats.** AT-RvD1 15 ng/50μl was directly applied onto the exposed spinal cord after stable baseline responses were established. The inhibitory effects on C-fibre and post-discharge (PD) responses peaked at 15 min post application and returned toward control levels at 60 min. Aβ fibre responses remained comparable to the control level throughout the hour. n=9 neurones.

**Figure S4 An explanation of input and wind-up (WU).**

The graph illustrates responses in C-fibre and post discharge bands (90-800 ms post stimulus) of a WDR neurone following a train of 16-electrical stimulation (0.5 Hz, 2 ms pulse width at 3-times C-fibre threshold). If there is no potentiation, the responses will be flat, shown as the orange theoretical line. The input is calculated by taking the initial response (10) multiply by stimulus number (16) which results in 160. The input represents initial or non-potentiated response of neurones. However, in the actual experiment, a WDR neurone can display an increase in responsiveness after repetitive stimulation (shown as red line) which is a typical characteristic. This phenomenon is called wind-up (WU) which represents enhanced excitability. The cumulative number of action potentials evoked by the train of stimulation (490) minus input (160) results in calculated WU 330.
